# Maternal mental health at 5 years and childhood overweight or obesity at 11 years: evidence from the UK Millennium Cohort Study

**DOI:** 10.1038/s41366-018-0252-5

**Published:** 2018-11-21

**Authors:** Steven Hope, Nadia Micali, Jessica Deighton, Catherine Law

**Affiliations:** 1 Population, Policy and Practice Programme, UCL Great Ormond Street Institute of Child Health, 30 Guilford Street, London, WC1N IEH UK; 2 UCL and Anna Freud National Centre for Children and Families, 12 Maresfield Gardens, London, NW3 5SU UK

**Keywords:** Risk factors, Health policy

## Abstract

**Background/objectives:**

Maternal psychological distress is associated with a range of adverse child outcomes. We sought to determine whether children’s exposure to medium or severe distress at 5 years was associated with increased risks of overweight and obesity when they were aged 11 years. We also investigated whether any association was attenuated after accounting for potential confounding and mediating factors.

**Subjects/methods:**

We analysed data from the UK Millennium Cohort Study, a nationally representative sample with data collected throughout childhood, imputing missing covariates (analytic sample: *n* = 9206). Multinomial regression was used to examine whether maternal psychological distress (Kessler-6 scale, using medium and severe score thresholds) at 5 years of age predicted children’s objectively measured overweight and obesity at 11 years, adjusting for sex and ethnicity. We then carried out a series of models incorporating potential confounders (early life and socio-demographic, recorded at 9 months) and mediators (physical activity and dietary factors, at 7 years) in turn, and then simultaneously.

**Results:**

A third of mothers reported distress when their child was aged 5 years (29% medium; 4% severe distress), and over a quarter of children were overweight at 11 years (22% overweight; 6% obese). Risks of obesity at 11 years increased with severity of maternal distress at 5 years (medium distress: relative risk ratio (RRR) = 1.43, 95% confidence interval [CI] 1.17–1.75; severe RRR = 2.27, CI 1.42–3.63). Adjusting for each set of explanatory factors in turn (particularly early years and socio-demographic confounding factors) reduced but did not eliminate these elevated risks. However, risks were attenuated in the fully adjusted model (medium: RRR = 1.14, CI 0.92–1.41; severe: RRR = 1.26, CI 0.75–2.11).

**Conclusions:**

We demonstrated that maternal psychological distress, particularly if severe, at 5 years was associated with risk of obesity (but not overweight) at 11 years. Accounting for potential explanatory factors attenuated this association to non-significance, suggesting a range of mechanisms may be implicated. Future research should seek to disentangle the potentially complex pathways linking explanatory factors, maternal distress and child obesity.

## Introduction

The increased prevalence of childhood overweight (including obesity) in high-income countries is a major public health concern, leading to greater risks of morbidity in childhood and adulthood [1]. The aetiology of childhood overweight is likely to be complex. However, identifying risk factors that are potentially amenable to change may provide opportunities to develop interventions aimed at reducing levels of overweight [2]. Poor maternal mental health is one such risk factor: it is common in the population (with a lifetime prevalence of experiencing a major depressive episode ranging between 6 and 17% for women, and higher rates of lower levels of distress [3]), and has been associated with a range of adverse outcomes in childhood [4, 5], including overweight [6, 7].

A number of mechanisms linking maternal mental health and child overweight have been proposed. There is conflicting evidence on possible early biological programming (resulting from distress in pregnancy or postpartum) increasing risks for overweight during childhood [8–10]. More direct influences on overweight may arise through a lack of parenting skills [11, 12], which may in turn affect health behaviours [13, 14], including unhealthy feeding practices and diet, lower levels of physical activity, increased sedentary behaviour and poorer sleep patterns [15–18]. However, confounding may also be in evidence, with socio-economic disadvantage being a risk both for poor maternal mental health and childhood overweight [6, 7], while predicting potential mediating factors, such as parenting, diet and physical activity [19, 20].

To date, there has been little longitudinal research, particularly investigating exposure to maternal mental health problems after infancy. Many studies have been cross-sectional and therefore temporality in the relationship between maternal mental health and overweight in childhood has not been established. Furthermore, research in this area has tended to rely on non-representative studies with small sample sizes, and few datasets have included the diverse range of potential mediators and confounders that may underlie this relationship. There is limited evidence differentiating exposure to medium and severe mental health problems [16, 17], or separating overweight from obesity in the outcome [11, 18, 21]. In addition, US samples have predominated, limiting the opportunity to understand this relationship in different national contexts.

Using longitudinal data from a contemporary UK child cohort (the Millennium Cohort Study, MCS) to capture temporality during the primary school period of childhood, we sought to determine whether exposure to medium or severe maternal psychological distress when the child was 5 years of age increased the risk of childhood overweight or obesity at 11 years. In addition, we used the rich data available in the MCS to account for a number of potential explanatory factors, including confounders (early years and socio-demographic characteristics) recorded at 9 months, and mediators (comprising dietary and physical activity behaviours) recorded at 7 years. Through a description of patterns of attenuation according to potential explanatory factors, we aimed to provide the basis for further research focusing on testing specific underlying causal pathways linking maternal psychological distress with childhood overweight and obesity.

## Methods

### Sample

The MCS is a large, contemporary cohort of UK children born between 2000 and 2002 which has been described elsewhere [22]. First contact with the cohort child and main respondent (usually the mother) occurred when the child was 9 months old, when information was collected on 18818 infants (of which we analysed 18296 singletons). At the time of carrying out these analyses, data were available for a further four sweeps, when the children were aged 3, 5, 7 and 11 years, each receiving approval from a Research Ethics Committee [23]. Data were obtained from the UK Data Archive, University of Essex in March 2014. This paper uses data from 10605 families who had taken part in the MCS at 9 months, 5 years, 7 years and 11 years, and where the main respondent was the mother. A total of 9206 children had complete data on exposure and outcome variables, of which 1286 children had missing data on one or more of the explanatory variables. We used multiple imputation by chained equations in 20 datasets to impute missing values for explanatory variables under a missing at random assumption.

### Measures

The temporality of exposure, outcome, and potential mediators and confounders were modelled using MCS data between 9 months and 11 years. Exposure, mediator and outcome variables were drawn from temporally ordered, consecutive sweeps of the MCS, reflecting the years during which the child was in primary school. The exposure was recorded at 5 years (start of primary school), potential mediators at 7 years, and the outcome at 11 years (the end of primary school). Potential confounders were measured at 9 months.

### Outcome: overweight or obesity

At 11 years, interviewers weighed the children, without shoes or outdoor clothing, using Tanita HD-305 scales (Tanita UK Ltd, Middlesex, UK). Weights were recorded in kilograms to one decimal place. Heights were obtained by the Leicester Height Measure Stadiometer (Seca Ltd, Birmingham, UK) and recorded to the nearest millimetre. Children were classified as overweight or obese using International Obesity Task Force (IOTF) international age- and sex-specific cut-offs for body mass index (BMI) [24].

### Exposure: maternal psychological distress

At 5 years, mothers completed a widely used and validated measure of psychological distress (the Kessler-6 scale, K-6) [25]. K-6 comprises six-items asking the mother how often during the past 30 days they felt: “so depressed that nothing could cheer you up”, “hopeless”, “restless or fidgety”, “that everything was an effort”, “worthless” and “nervous”. Each item had a five-point response, from “None of the time” (0) to “All of the time” (4). Responses to each item were combined to produce a single score ranging from 0 to 24. Using established cut-offs [26], the score was categorised as medium distress (K-6 score ≥  4) or severe distress (≥13).

### Explanatory factors

Factors that might influence the relationship between maternal distress at 5 years and child overweight at 11 years, either as potential confounders or mediators, were included in multivariable models. Variables that might potentially confound the relationship between maternal mental health and childhood overweight included socio-economic status [7] and factors relating to characteristics of the parents and the early life of the child [6]. Diet and physical activity are important predictors of childhood overweight [27], and have been shown to play a mediating role in the association between maternal mental distress and childhood overweight [15–18].

Potential explanatory factors (including categories) are shown in Table 1, and grouped in models as follows.

**Table 1:** Association between maternal psychological distress at 5 years and covariates at 9 months and 7 years with child overweight or obesity at 11 years (*n* = 9206)

	Child BMI status at 11 years							
	Normal BMI		Overweight		Obese		Total	
	%	95% CI	%	95% CI	%	95% CI	%	95% CI
Maternal psychological distress *p* < 0.001								
None/low	68.4	[66.9, 69.8]	65	[62.2, 67.8]	58	[53.3, 62.7]	67	[65.7, 68.3]
Medium	28.2	[26.9, 29.5]	31.9	[29.4, 34.5]	35	[30.7, 39.3]	29.4	[28.2, 30.6]
Severe	3.4	[2.9, 4.0]	3	[2.1, 3.9]	7	[4.3, 9.6]	3.6	[3.1, 4.1]
Child’s sex *p* < 0.001								
Male	53.1	[51.5, 54.7]	47	[43.9, 50.1]	49.6	[45.3, 53.9]	51.5	[50.1, 53.0]
Female	46.9	[45.3, 48.5]	53	[49.9, 56.1]	50.4	[46.1, 54.7]	48.5	[47.0, 50.0]
Child’s ethnicity *p* < 0.001								
White	90.9	[89.2, 92.5]	87.6	[84.7, 90.6]	86.2	[81.9, 90.5]	89.9	[89.0, 91.8]
Mixed	2.6	[0.2, 3.2]	3.4	[2.3, 4.4]	3.3	[1.6, 4.9]	2.8	[2.3, 3.3]
Indian	1.6	[1.0, 2.1]	1.9	[1.1, 2.7]	0.8	[0.0, 1.6]	1.6	[1.1, 2.1]
Pakistani or Bangladeshi	2.3	[1.4, 3.2]	2.9	[1.4, 4.4]	4	[1.9, 6.1]	2.5	[1.5, 3.5]
Black or Black British	1.9	[1.2, 2.7]	3.3	[1.7, 5.0]	5.5	[2.3, 8.6]	2.5	[1.4, 3.5]
Other	0.7	[0.6, 1.1]	0.8	[0.3, 1.3]	0.3	[−0.0, 0.5]	0.7	[0.4, 1.0]
Early years factors								
Maternal BMI before birth of child *p* < 0.001								
BMI (means)	23.1	[22.9, 23.2]	25.1	[24.8, 25.5]	27.4	[26.8, 27.9]	23.8	(23.6, 23.9)
Mother smoked during pregnancy *p* < 0.001								
No	65.8	[64.1, 67.6]	60.4	[57.7, 63.1]	54	[48.8, 59.2]	63.9	[62.4, 65.5]
Yes	34.2	[32.4, 35.9]	39.6	[36.9, 42.3]	46	[40.8, 51.2]	36.1	[34.5, 37.6]
Child birthweight *p* < 0.001								
Birthweight (*z* score, means)	−0.07	[−.10, −.04]	0.04	[−.02, .10]	0.06	[−.04, .17]	−0.04	[−.07, −.01]
Duration of breastfeeding *p* < 0.001								
Never	30.8	[28.6, 33.0]	34.9	[31.9, 37.9]	42.7	[37.3, 48.0]	32.4	[30.4, 34.5]
1 week	11.1	[10.0, 12.2]	12	[10.4, 13.6]	12.3	[9.2, 15.3]	11.4	[10.4, 12.3]
1–6 weeks	12.8	[11.8, 13.8]	12.5	[10.7, 14.3]	14	[10.5, 17.5]	12.8	[11.9, 13.7]
6 weeks − 4 months	16.5	[15.3, 17.7]	15.5	[13.6, 17.5]	14.3	[11.0, 17.6]	16.1	[15.1, 17.2]
Over 4 months	28.8	[26.6, 31.0]	25.1	[22.6, 27.6]	16.8	[13.1, 20.5]	27.2	[25.3, 29.2]
Child introduced to solids before 4 months *p* < 0.001								
No	65	[63.3, 66.7]	61.2	[58.7, 63.7]	54.6	[49.4, 59.8]	63.5	[62.1, 65.0]
Yes	35	[32.4, 35.9]	38.8	[36.3, 41.3]	45.4	[40.2, 50.6]	36.5	[35.0, 37.9]
Socio-demographic factors								
Maternal highest qualification (9 months) *p* < 0.001								
A/AS/S levels and above	40.9	[38.2, 43.6]	34.2	[31.2, 37.2]	23.5	[19.2, 27.8]	38.4	[36.0, 40.8]
O levels/GCSEs A-C	36.1	[34.1, 38.1]	39.5	[36.9, 42.1]	36	[31.6, 40.5]	36.9	[35.2, 38.6]
O levels/GCSEs D-G and below	23	[21.2, 24.8]	26.2	[23.8, 28.7]	40.5	[35.3, 45.6]	24.8	[23.1, 26.5]
Family structure (9 months) *p* < 0.01								
Couple family	86.2	[85.0, 87.4]	84.5	[82.3, 86.8]	80	[76.0, 83.9]	85.4	[84.3, 86.6]
Lone parent family	13.8	[12.6, 15.0]	15.5	[13.2, 17.7]	20	[16.1, 24.0]	14.6	[13.4, 15.7]
Household income (9 months) *p* < 0.001								
Lowest quintile	18.1	[16.4, 19.8]	20.6	[18.0, 23.3]	26	[21.5, 30.5]	19.1	[17.5, 20.8]
Second quintile	18.5	[17.0, 20.0]	22.7	[20.3, 25.2]	27.5	[23.2, 31.8]	20	[18.6, 21.4]
Third quintile	20.6	[19.1, 22.1]	21	[18.8, 23.1]	20.2	[16.4, 24.1]	20.6	[19.4, 21.9]
Fourth quintile	21	[19.6, 22.4]	20.7	[18.5, 23.0]	17.1	[13.6, 20.6]	20.7	[19.5, 22.0]
Highest quintile	21.8	[19.2, 24.4]	14.9	[12.9, 16.9]	9.2	[6.6, 11.8]	19.5	[17.3, 21.7]
Housing tenure (9 months) *p* < 0.001								
Mortgage/own outright	64.7	[62.4, 66.9]	57.8	[54.7, 61.0]	50.9	[45.8, 55.9]	62.3	[60.2, 64.5]
Private rental	8	[6.8, 9.3]	8.6	[6.9, 10.2]	7.4	[4.5, 10.4]	8.1	[7.0, 9.2]
Local authority/housing assoc. rental	22.5	[20.4, 24.6]	27	[24.0, 30.0]	34.9	[29.8, 40.0]	24.2	[22.2, 26.3]
Other	4.8	[4.1, 5.5]	6.6	[5.1, 8.2]	6.8	[4.3, 9.3]	5.3	[4.6, 6.0]
Dietary factors								
Portions of fruit consumed by child/typical day (7 years) *p* < 0.001								
3 or more	54.9	[53.1, 56.7]	52.3	[49.5, 55.0]	44.1	[39.1, 49.1]	53.6	[52.1, 55.2]
Two	25.1	[23.8, 26.4]	26.4	[24.1, 28.7]	29.1	[24.7, 33.5]	25.6	[24.5, 26.8]
One or none	20	[18.7, 21.4]	21.3	[18.9, 23.8]	26.8	[22.7, 31.0]	20.7	[19.5, 22.0]
Breakfast consumption/week (7 years) *p* < 0.001								
0–3 days	2.1	[1.6, 2.6]	3.4	[2.4, 4.3]	5.8	[3.5, 8.1]	2.6	[2.2, 3.1]
4–6 days	3	[2.5, 3.4]	4.1	[3.0, 5.2]	5.7	[3.7, 7.6]	3.4	[2.9, 3.8]
7 days	94.9	[94.3, 95.6]	92.5	[91.0, 94.0]	88.5	[85.8, 91.2]	94	[93.4, 94.6]
Main drinks between meals (7 years) *p* < 0.001								
Other drinks	58.4	[56.0, 60.7]	55	[52.1, 57.9]	49.8	[45.4, 55.1]	57.1	[55.0, 59.2]
Sweetened drinks	41.6	[39.3, 44.0]	45	[42.1, 47.9]	50.2	[44.9, 55.6]	42.9	[40.8, 45.0]
Main snack between meals (7 years) *p* < 0.001								
Crisps and other similar snacks	14.2	[13.0, 15.3]	17.8	[15.6, 19.9]	22.2	[18.0, 26.5]	15.5	[14.5, 16.4]
Cakes and sweet biscuits	17.5	[16.2, 18.8]	13.1	[11.2, 15.1]	16.4	[12.9, 20.0]	16.5	[15.4, 17.5]
Fruit or vegetables	40.7	[39.2, 42.3]	39.2	[36.3, 42.0]	33	[28.4, 37.5]	39.9	[38.6, 41.2]
Bread and similar items	7.8	[6.8, 8.7]	7.8	[6.3, 9.3]	8.4	[5.8, 11.0]	7.8	[7.0, 8.7]
Sweets or chocolate	8.6	[7.7, 9.5]	8.3	[6.8, 9.8]	8.9	[6.3, 11.5]	8.6	[7.8, 9.3]
Dairy products	9.3	[8.5, 10.2]	11.9	[10.0, 13.8]	9	[5.8, 12.2]	9.9	[9.1, 10.6]
Does not eat between meals	1.8	[1.5, 2.2]	1.9	[1.1, 2.7]	2	[0.7, 3.4]	1.9	[1. 5, 2.2]
Physical activity factors								
Number of days/week child participated in organised sports/activities (7 years) *p* < 0.001								
3 or more days/week	23.3	[21.7, 24.9]	18.7	[16.5, 20.8]	12.8	[9.6, 15.9]	21.6	[20.3, 23.0]
2 days/week	22.4	[21.0, 23.7]	21.9	[19.7, 24.1]	18.3	[14.8, 21.9]	22	[20.9, 23.2]
1 day/week	26.4	[25.1, 27.8]	27.7	[25.1, 30.2]	26.7	[22.6, 30.8]	26.7	[25.6, 27.9]
Less often or not at all	27.9	[25.8, 30.0]	31.7	[28.9, 34.6]	42.2	[37.2, 47.2]	29.6	[27.8, 31.4]
Number of days/week child participated in non-organised physical activity (7 years) *p* < 0.05								
3 or more days/week	81.4	[80.0, 82.8]	79.3	[76.8, 81.8]	76	[71.8, 80.1]	80.6	[79.3, 81.9]
2 days/week	7.9	[7.1, 8.7]	8.4	[6.9, 9.9]	7.7	[5.3, 10.1]	8	[7.3, 8.7]
1 day/week	5.4	[4.7, 6.0]	6.2	[4.9, 7.5]	7.3	[4.9, 9.7]	5.7	[5.1, 6.3]
Less often or not at all	5.3	[4.5, 6.1]	6.1	[4.7, 7.5]	9	[6.2, 11.8]	5.7	[4.9, 6.4]
Frequency mother participated in active play with child (7 years) *p* < 0.01								
Weekly	51.3	[49.7, 52.9]	46.8	[43.9, 49.7]	45.5	[40.3, 50.7]	50	[48.6, 51.3]
Once or twice a month	21.8	[20.6, 23.0]	21.9	[19.8, 24.1]	21.4	[17.3, 25.4]	21.8	[20.8, 22.8]
Less often than once a month	15	[14.1, 15.8]	16.4	[14.5, 18.3]	15.4	[12.0, 18.7]	15.3	[14.5, 16.1]
Not at all	12	[10.9, 13.0]	14.9	[12.9, 17.0]	17.8	[13.4, 22.1]	13	[12.0, 13.9]
Transport to school (7 years) *p* = 0.581								
Motorised	49.3	[47.6, 51.1]	49.6	[47.1, 52.1]	46.7	[41.7, 51.7]	49.2	[47.7, 50.8]
Active (Walking/ cycling)	50.7	[48.9, 52.4]	50.4	[47.9, 52.9]	53.3	[48.3, 58.3]	50.8	[49.2, 52.3]
Regular weekday term-time bedtime (7 years) *p* < 0.001								
Before 19:30	11.7	[10.6, 12.8]	8.6	[7.0, 10.3]	9	[6.1, 11.9]	10.8	[9.9, 11.8]
19:30–20:00	25.9	[24.3, 27.4]	20.7	[18.5, 23.0]	18.5	[14.5, 22.5]	24.3	[23.0, 25.6]
20:00–20:30	35.4	[33.9, 36.8]	36.6	[34.0, 39.1]	32.7	[28.1, 37.2]	35.5	[34.2, 36.7]
20:30–21:00	14.4	[13.3, 15.5]	17.6	[15.5, 19.6]	15.7	[12.5, 19.0]	15.2	[14.2, 16.2]
21:00+	9.1	[8.2, 10.0]	13.2	[11.5, 15.0]	17.1	[13.2, 21.1]	10.5	[9.6, 11.4]
No regular bedtime	3.6	[3.0, 4.1]	3.3	[2.3, 4.2]	7	[4.6, 9.4]	3.7	[3.3, 4.2]
Number of hours a day child watched television/videos (7 years) *p* < 0.001								
None or up to 1 h	21.3	[19.7, 22.9]	16.6	[14.7, 18.6]	14.7	[11.1, 18.2]	19.8	[18.5, 21.1]
More than 1 h, less than 3 h	64.6	[63.0, 66.3]	65.9	[63.5, 68.3]	64.9	[59.9, 70.0]	64.9	[63.6, 66.3]
3 h or more	14.1	[12.6, 15.6]	17.5	[15.5, 19.5]	20.4	[16.4, 24.4]	15.2	[14.0, 16.5]
Number of hours a day child used computer (7 years) *p* < 0.01								
None or up to 1 h	65.4	[63.7, 67.1]	62.6	[60.0, 65.3]	58.8	[54.1, 63.5]	64.4	[62.8, 65.9]
More than 1 h, less than 3 h	30.5	[28.9, 32.1]	33.7	[31.1, 36.4]	36.5	[31.9, 41.0]	31.6	[30.1, 33.0]
3 h or more	4.1	[3.4, 4.8]	3.7	[2.7, 4.6]	4.7	[2.7, 6.8]	4.1	[3.4, 4.7]

#### Early years

Maternal pre-pregnancy body size (self-reported pre-pregnancy weight and current height, converted to BMI), whether the mother smoked in pregnancy, child’s birthweight (converted to *z*-scores, adjusting for sex and gestational age), duration of breastfeeding, and whether the child had been introduced to solids before 4 months of age. All early years variables were recorded at 9 months.

#### Socio-demographics

Maternal highest academic qualification, family structure, and household income quintiles (calculated using a modified OECD equivalence scale). For the main analysis, all variables were recorded at 9 months. However, family structure and income are potentially time-varying factors. As a sensitivity analysis, analyses were repeated using values from 7 years (the sweep between exposure and outcome).

Child health behaviours that might mediate the relationship between maternal distress at 5 years and child overweight at 11 years were selected from 7-year maternal report.

#### Diet

Maternal report of cohort child’s daily consumption of portions of fruit, number of days per week breakfast consumed, consumption of sweetened drinks between meals, and snacks between meals.

#### Physical activity

Maternal report about frequency the child participated in organised sports or activities, non-organised sports or activities, active play between mother and child, means of transport to school, regularity and timing of bedtime, and number of hours per day the child watched television or videos and used a computer.

Multivariable models also included sex and ethnicity of the cohort child.

### Statistical analysis

All analyses were conducted in Stata/SE 13 (Stata Corporation, TX), using survey and non-response weights to account for clustered sampling design and attrition up to 11 years. We calculated percentages (or means) for exposure, explanatory and outcome variables, overall and by maternal distress and child overweight. We used multinomial logistic regression models to estimate relative risk ratios and 95% confidence intervals (CIs) for childhood weight (normal, overweight, or obese) at 11 years according to maternal psychological distress when the child was 5 years old. There was no interaction between sex of the child and maternal distress and therefore analyses were carried out for boys and girls combined. Analyses were carried out using an imputed sample (*n* = 9206). A sensitivity analysis, which included in the final model a marker of puberty (maternal report at 11 years of whether the child was reported to have experienced a growth spurt), did not alter the association between maternal distress and overweight status, either as a main effect or interacting with gender.

The models tested were as follows:

Model A: Maternal psychological distress + child’s sex and ethnicity

Model B: A + early years factors

Model C: A + socio-demographic factors

Model D: A + dietary factors

Model E: A + physical activity factors

Model F: Fully adjusted for explanatory factors

## Results

### Descriptive statistics

A third of mothers (33%) reported distress when their child was aged 5 years, at predominantly medium (29%) rather than severe (4%) levels. At 11 years of age, most cohort children (72%) were within the normal weight range, with more than a quarter of the children either overweight (22%) or obese (6%). Table 1 shows the relationship between each of the explanatory factors and BMI weight status at 11 years. Both overweight and obesity at 11 years were predicted by early years and socio-demographic factors recorded at 9 months of age and by dietary and physical activity factors (with the exception of means of transport to school) at 7 years, although relationships were stronger for obese children. Compared to children who were normal weight, children who were obese were more likely to have had: a mother with a higher pre-pregnancy BMI, a mother who smoked during pregnancy, a higher birthweight, no or a shorter period of breastfeeding, and an early introduction to solids. Obese children were also more likely to have been brought up in less advantaged socio-economic circumstances and to have experienced adverse health behaviours at 7 years (lower fruit consumption, not having a regular breakfast, greater sweetened drink consumption, snacking on crisps, less sport or physical activity, irregular or late bedtimes, and higher levels of screentime).

Most explanatory factors were also associated with maternal distress at 5 years (Supplementary Table S1), including early factors (except child’s sex and maternal pre-pregnancy BMI), adverse socio-economic circumstances and adverse health behaviours at 7 years. Risks were particularly elevated if the mother had reported severe distress.

### Regression models

Risk of overweight at 11 years was significantly raised in children whose mothers reported medium levels of psychological distress at 5 years, after accounting for sex and ethnicity (Table 2: Model A). However, this association was attenuated to non-significance in all subsequent models. The association between maternal distress and obesity was stronger than for overweight, with raised risks for medium and, particularly, severe distress (Model A). Adjusting for early years factors (Model B) and socio-demographic factors at 9 months of age (Model C) attenuated the association between maternal distress and child obesity, although it remained elevated for both medium and severe distress. The remaining models accounted for potential mediators between maternal distress and child obesity through child health behaviours recorded at 7 years. As with the early factors, adjusting for dietary (Model D) and physical activity factors (Model E) attenuated the association between maternal distress and child obesity, although risks remained elevated. Finally, the association between maternal distress and obesity was attenuated to non-significance for both medium and severe levels of distress when all risk factors were accounted for simultaneously (Table 2: Model F). Sensitivity analyses carried out with socio-demographic characteristics recorded at 7 years produced similar results (not shown), reflecting continuity in the socio-economic circumstances of children’s families.

**Table 2:** Relative risk ratios (95% confidence intervals) for the relationship between maternal psychological distress at cohort child age 5 years and child overweight or obesity at 11 years (*n* = 9 206)

	% (*n*) maternal psychological distress 5 y and weight at 11 y	Adjusted for sex of child, ethnicity	Model A + Early years factors (9 m)	Model A + Socio-demographic factors (9 m)	Model A + dietary factors (7 y)	Model A + physical activity factors (7 y)	Fully adjusted
		(A)	(B)	(C)	(D)	(E)	(F)
Normal range							
No distress	73.4 (4591)	**—**	—	—	—	—	—
Medium distress	68.9 (1791)						
Severe distress	69.4 (204)						
Overweight							
No distress	21.2 (1361)						
Medium distress	23.7 (601)	1.18 (1.02–1.35)	1.11 (0.97–1.28)	1.11 (0.96–1.27)	1.14 (0.99–1.31)	1.14 (0.99–1.31)	1.07 (0.92–1.24)
Severe distress	18.6 (60)	0.89 (0.63–1.27)	0.75 (0.52–1.09)	0.78 (0.55–1.12)	0.81 (0.57–1.14)	0.85 (0.60–1.20)	0.70 (0.49–1.00)
Obese							
No distress	5.3 (364)						
Medium distress	7.3 (199)	1.43 (1.17–1.75)	1.26 (1.02–1.55)	1.25 (1.02–1.53)	1.35 (1.10–1.66)	1.29 (1.06–1.58)	1.14 (0.92–1.41)
Severe distress	12.0 (35)	2.27 (1.42–3.63)	1.56 (0.94–2.60)	1.64 (1.00–2.69)	1.83 (1.15–2.93)	1.85 (1.16–2.97)	1.26 (0.75–2.11)

A: Maternal distress, adjusted for cohort child’s sex and ethnicity

B: Model A + early years factors (duration maternal smoking in pregnancy; duration breastfeeding; introduction to solids; birthweight; maternal pre-pregnancy BMI)

C: Model A + Socio-demographic factors at 9 months (maternal highest academic qualification; family structure; household income quintiles; housing tenure)

D: Model A + dietary factors (fruit consumption; breakfast consumption; sweetened drinks between meals; main snack between meals)

E: Model A + physical activity factors (child participated in sport/PA, child non-organised physical activity, mother engaged in active play with child, transport to school, regular bedtime, TV screentime hours, computer screentime hours)

F: Fully adjusted (Model A + Models B, C, D and E)

## Discussion

### Summary of findings

In a nationally representative contemporary sample of UK children, maternal distress and childhood overweight were common. We used longitudinal data to demonstrate that maternal psychological distress at 5 years was associated with increased risks of obesity at 11 years. This was demonstrated for both medium and severe levels of distress, although severe distress resulted in the greater risk of obesity. Within the MCS, we identified an array of explanatory factors that might confound or mediate the relationship between maternal mental health and obesity. Perinatal and infancy factors, socio-demographic characteristics, and indicators of the child’s diet and physical activity were associated with maternal distress at 5 years and child overweight or obesity status at 11 years. Each set of explanatory factors attenuated the association between maternal distress and childhood obesity to some extent, although only after all potential explanatory factors were accounted for simultaneously were the risks associated with both medium and severe maternal distress attenuated to non-significance.

### Comparison with other findings

The evidence linking maternal mental health problems with childhood overweight and obesity is inconsistent, with some studies finding that an association exists [6, 7, 28] while others have reported no such association [8, 9, 21]. In accord with some of these studies, we showed a stronger association between maternal distress and obesity (rather than overweight) [11, 18, 28]. We identified risks for both medium and severe levels of distress, albeit that these risks were greater for severe distress. Possible mechanisms linking distress with childhood weight have included parenting skills, family feeding practices, and a more sedentary lifestyle [11–18]. Furthermore, the child’s own mental health may influence their health behaviours and, in turn, increase their risk of overweight or obesity, as has been shown for children with ADHD [29] or anxiety [30]. In addition, there is a potentially complex interplay between maternal distress and child mental health, whereby the mental health problems of the child may increase the mother’s distress [31]. Unlike the majority of previous research, we were able to model a plausible temporal sequence between exposure and outcome, adjusting for confounders measured early in the child’s life and mediators at the intermediate data collection sweep. However, ours was an exploratory study, which aimed to identify areas that would benefit from further research.

### Strengths and limitations

A major strength of these analyses is the use of the UK Millennium Cohort Study, a large, nationally representative contemporary cohort. Heights and weights of the children were recorded objectively by interviewers. We used these data to construct a measure of BMI with IOTF cut-offs (for overweight and obesity). This measure is commonly used in population research, and provides internationally comparable results. In addition, it is relevant for and interpretable by policymakers. Nevertheless, it would have been informative to also have a measure of central adiposity; unfortunately, this is not available in the MCS. The MCS has detailed information on the early life and socio-demographic characteristics of the cohort child and their family. However, measures of diet and physical activity available were relatively crude, and there was no suitable measure of parenting available at 7 years (as a potential mediator between distress and overweight). Maternal distress was measured using the Kessler-6 scale, a widely used, validated scale. Nevertheless, the K-6, and all explanatory variables were completed by the mother, carrying the potential for report bias. Attrition is always a concern in longitudinal research. Mothers who were identified as having medium or severe psychological distress at 5 years, or if a child was identified as obese at 11 years, were less likely to have complete data for the analyses. We used multiple imputation and response weights [32] to account for attrition and item missingness, although results may still have been subject to bias due to these factors. Finally, despite our focus on maternal distress we acknowledge that risks of childhood overweight and obesity might also be influenced by paternal factors. Nevertheless, mothers are likely to have been the main carers for children in the period from early to mid-childhood [33, 34], and data on fathers have not been well-measured in the MCS.

### Implications for policy and research

These results show that maternal distress recorded at the start of a child’s schooling predicted subsequent obesity in mid-childhood and suggest that multifaceted interventions should be considered for families where the mother is experiencing distress. Maternal distress is likely to be linked to the mother’s parenting capacities, food and exercise practices, and wider family and social circumstances. In addition, mothers with mental health problems are more likely themselves to be obese [35]. Factors representing a range of influences, including early years, socio-demographic circumstances, and dietary and physical activity behaviours were implicated in the development of the association between maternal distress and child obesity over time in the MCS. Our research has provided a description of diverse influences underlying the relationship between maternal distress and child obesity. Although we were not testing causal pathways, our findings suggest that no dominant single mechanism or set of explanatory factors can account for the association between maternal distress and child obesity in mid-childhood. This is consistent with a socio-ecological perspective, acknowledging the multifaceted influences that lead to the development of childhood obesity. This study highlights a number of explanatory factors on which it would be potentially useful to carry out further research. This might include developing and testing causal models to explicitly identify and test particular confounding and mediating pathways linking maternal distress and childhood obesity, including the interplay between maternal and child mental health.

## Electronic supplementary material

Supplementary Table S1
