# Supplementary material for: Maternal mental health at 5 years and childhood overweight or obesity at 11 years: evidence from the UK Millennium Cohort Study
Source: Int J Obes (Lond). 2018 Nov 21;43(1):43–52. doi: 10.1038/s41366-018-0252-5 (PMC6331386; doi:10.1038/s41366-018-0252-5)
Supplement: Supplementary file 1 — Supplementary Table S1 [file 41366_2018_252_MOESM1_ESM.docx]

**SUPPLEMENTARY TABLE S1: Associations between maternal psychological distress at 5 years, childhood overweight or obesity at 11 years and covariates at 9 months and 7 years (n=9 206)**

|  | **Maternal psychological distress at 5 years** | | | | | | | |
| --- | --- | --- | --- | --- | --- | --- | --- | --- |
|  | **no/low distress** | | **medium distress** | | **severe distress** | | **Total** | |
|  | **%** | **CI** | **%** | **CI** | **%** | **CI** | **%** | **CI** |
| **Child BMI status at 11y** *p<0.001* | | | | | | | | |
| Normal BMI | 73.4 | [72.0,74.9] | 68.9 | [66.9,71.0] | 69.4 | [63.5,75.3] | 72.0 | [70.8,73.1] |
| Overweight | 21.2 | [19.9,22.5] | 23.7 | [21.7,25.8] | 18.6 | [13.4,23.7] | 21.9 | [20.8,23.0] |
| Obese | 5.3 | [4.7,6.0] | 7.3 | [6.3,8.4] | 12.0 | [7.5,16.6] | 6.2 | [5.6,6.7] |
| **Child’s sex**  *p=0.630* | | | | | | | | |
| Male | 51.2 | [49.8,52.7] | 52.4 | [49.8,55.0] | 49.9 | [42.7,57.1] | 51.5 | [50.1,53.0] |
| female | 48.8 | [47.3,50.2] | 47.6 | [45.0,50.2] | 50.1 | [42.9,57.3] | 48.5 | [47.0,50.0] |
| **Child’s ethnicity**  *p<0.001* | | | | | | | | |
| White | 91.7 | [90.1,93.3] | 86.5 | [83.6,89.4] | 83.2 | [77.1,89.3] | 90.0 | [88.0,91.8] |
| Mixed | 2.7 | [2.1,3.3] | 3.1 | [2.3,3.8] | 3.9 | [1.2,6.6] | 2.8 | [2.3,3.3] |
| Indian | 1.4 | [1.0,2.0] | 1.9 | [1.1,2.6] | 2.2 | [0.5,3.8] | 1.6 | [1.1,2.1] |
| Pakistani or Bangladeshi | 1.8 | [1.1,2.6] | 3.8 | [2.1,5.5] | 5.4 | [0.6,10.2] | 2.5 | [1.5,3.5] |
| Black or Black British | 1.9 | [1.0,2.8] | 3.4 | [1.9,5.0] | 4.6 | [1.3,7.9] | 2.5 | [1.4,3.5] |
| Other | 0.4 | [0.2,0.6] | 1.4 | [0.5,2.2] | 0.7 | [-.0,1.5] | 0.7 | [0.4,1.0 |
| ***EARLY YEARS FACTORS*** | | | | | | | | |
| **Maternal BMI before birth of child**  *p=0.097* | | | | | | | | |
| BMI (means) | 23.7 | [23.6,23.9] | 23.8 | [23.6,24.1] | 24.3 | [23.4,25.2] | 23.8 | [23.6,23.9] |
| **Mother smoked during pregnancy**  *p<0.001* | | | | | | | | |
| No | 68.3 | [66.7,70.0] | 56.4 | [54.0,58.8] | 43.5 | [36.9,50.1] | 63.9 | [62.4,65.5] |
| Yes | 31.7 | [30.0,33.3] | 43.6 | [41.2,46.0] | 56.5 | [49.9,63.1] | 36.1 | [34.5,37.6] |
| **Child birthweight**  *p<0.001* | | | | | | | | |
| birthweight (z score, means) | -.00 | [-.03,.03] | -.11 | [-.16,-.06] | -.18 | [-.34,-.01] | -.04 | [-.07,-.01] |

|  | **Maternal psychological distress at 5 years** | | | | | | | | | | |
| --- | --- | --- | --- | --- | --- | --- | --- | --- | --- | --- | --- |
|  | **no/low distress** | | **medium distress** | | | | **severe distress** | | | **Total** | |
|  | **%** | **CI** | **%** | | **CI** | | **%** | | **CI** | **%** | **CI** |
| **Duration of breastfeeding**  *p<0.001* | | | | | | | | | | | |
| Never | 30.8 | [28.7,32.8] | 35.0 | [31.9,38.0] | | 42.9 | | | [35.1,50.7] | 32.4 | [30.4,34.5] |
| 1 week | 11.5 | [10.5,12.5] | 11.1 | [9.6,12.7] | | 10.5 | | | [6.8,14.2] | 11.4 | [10.4,12.3] |
| 1-6 weeks | 12.5 | [11.5,13.5] | 13.2 | [11.4,15.0] | | 15.3 | | | [10.3,20.3] | 12.8 | [11.9,13.7] |
| 6 weeks - 4 months | 16.0 | [14.9,17.1] | 16.5 | [14.6,18.4] | | 16.2 | | | [10.7,21.7] | 16.1 | [15.1,17.2] |
| over 4 months | 29.2 | [27.1,31.4] | 24.2 | [21.8,26.6] | | 15.0 | | | [10.3,19.8] | 27.2 | [25.3,29.2] |
| **Child introduced to solids before 4 months**  *p<0.05* | | | | | | | | | | | |
| No | 63.7 | [62.0,65.3] | 64.3 | [61.9,66.6] | | 55.0 | | | [48.0,62.0] | 63.5 | [62.1,65.0] |
| Yes | 36.3 | [34.7,38.0] | 35.7 | [33.4,38.1] | | 45.0 | | | [38.0,52.0] | 36.5 | [35.0,37.9] |
| ***SOCIO-DEMOGRAPHIC FACTORS*** | | | | | | | | | | | |
| **Maternal highest qualification (9 months)** *p<0.001* | | | | | | | | | | | |
| A/AS/S levels and above | 42.1 | [39.4,44.7] | 32.9 | [30.0,35.8] | | 13.7 | | | [9.2,18.2] | 38.4 | [36.0,40.8] |
| O levels/GCSEs A-C | 36.7 | [34.6,38.8] | 37.2 | [34.8,39.5] | | 37.3 | | | [30.8,43.8] | 36.9 | [35.1,38.6] |
| O levels/GCSEs D-G and below | 21.2 | [19.6,22.8] | 29.9 | [27.1,32.8] | | 49.0 | | | [41.9,56.1] | 24.8 | [23.1,26.5] |
| **Family structure (9 months)**  *p<0.001* | | | | | | | | | | | |
| Couple family | 88.2 | [87.1, 89.4] | 80.6 | [78.4,82.7] | | 73.1 | | | [66.7,79.6] | 85.4 | [84.3,86.6] |
| Lone parent family | 11.8 | [10.6,12.9] | 19.4 | [17.3,21.6] | | 26.9 | | | [20.4,33.3] | 14.6 | [13.4,15.7] |
| **Household income (9 months)** *p<0.001* | | | | | | | | | | | |
| Lowest quintile | 15.1 | [13.7,16.5] | 25.4 | | [22.6,28.3] | | 42.5 | | [35.6,49.4] | 19.1 | [17.5,20.8] |
| Second quintile | 17.9 | [16.4,19.4] | 23.7 | | [21.5,25.9] | | 28.9 | | [23.4,34.5] | 20.0 | [18.6,21.4] |
| Third quintile | 20.7 | [19.2,22.2] | 20.9 | | [18.7,23.1] | | 17.0 | | [11.3,22.6] | 20.6 | [19.4,21.9] |
| Fourth quintile | 23.2 | [21.8,24.7] | 16.5 | | [14.7,18.3] | | 8.3 | | [4.6,11.9] | 20.7 | [19.5,22.0] |
| Highest quintile | 23.0 | [20.4,25.6] | 13.4 | | [11.4,15.5] | | 3.3 | | [0.9,5.7] | 19.5 | [17.3,21.7] |
| **Housing tenure (9 months)** *p<0.001* | | | | | | | | | | | |
| Mortgage / own outright | 69.0 | [66.8,71.2] | 50.9 | | [48.0,53.8] | | 31.0 | | [25.0,37.0] | 62.3 | [60.2,64.5] |
| Private rental | 7.2 | [6.1,8.4] | 9.4 | | [8.0,10.9] | | 13.6 | | [8.8,18.4] | 8.1 | [7.0,9.2] |
| Local authority/housing assoc. rental | 18.9 | [16.9,20.8] | 33.3 | | [30.4,36.3 | | 50.5 | | [43.0,57.9] | 24.2 | [22.2,26.3] |
| Other | 4.9 | [4.2,5.6] | 6.3 | | [5.0,7.6] | | 4.9 | | [2.2,7.7] | 5.3 | [4.6,6.0] |
|  | **Maternal psychological distress at 5 years** | | | | | | | | | | |
|  | **no/low distress** | | **medium distress** | | | | **severe distress** | | | **Total** | |
|  | **%** | **CI** | **%** | | **CI** | | **%** | | **CI** | **%** | **CI** |
| ***DIETARY FACTORS*** | | | | | | | | | | | |
| **Portions of fruit consumed by child / typical day (7 years)**  *p<0.001* | | | | | | | | | | | |
| 3 or more | 55.8 | [54.1,57.4] | 50.8 | | [48.0,53.6] | | 37.1 | | [30.6,43.7] | 53.6 | [52.1,55.2] |
| Two | 25.2 | [24.0,26.4] | 26.2 | | [23.8,28.6] | | 28.9 | | [22.8,35.1] | 25.6 | [24.5,26.8] |
| one or none | 19.0 | [17.8,20.3] | 23.0 | | [20.7,25.2] | | 33.9 | | [27.3,40.5] | 20.7 | [19.5,22.0] |
| **Breakfast consumption /week (7 years)**  *p<0.001* | | | | | | | | | | | |
| 0-3 days | 2.0 | [1.6,2.3] | 3.5 | | [2.5,4.5] | | | 8.1 | [3.8,12.5] | 2.6 | [2.2,3.1] |
| 4-6 days | 2.7 | [2.2,3.2] | 4.6 | | [3.6,5.6] | | | 6.1 | [2.8,9.3] | 3.4 | [2.9,3.8] |
| 7 days | 95.3 | [94.8,95.9] | 91.9 | | [90.5,93.4] | | | 85.8 | [80.7,90.9] | 94.0 | [93.4,94.6] |
| **Main drinks between meals (7 years)**  *p<0.01* | | | | | | | | | | | |
| Other drinks | 58.2 | [56.0,60.4] | 56.2 | | [52.9,59.5] | | | 44.1 | [37.4,50.8] | 57.1 | [55.0,59.2] |
| Sweetened drinks | 41.8 | [39.6,44.0] | 43.8 | | [40.5,47.1] | | | 55.9 | [49.2,62.6] | 42.9 | [40.8,45.0] |
| **Main snack between meals (7 years)** *p<0.001* | | | | | | | | | | | |
| crisps and other similar snacks | 13.9 | [12.8,15.0] | 17.8 | | [15.9,19.6] | | | 25.9 | [19.7,32.2] | 15.5 | [14.5,16.4] |
| cakes and sweet biscuits | 16.9 | [15.7,18.1] | 16.1 | | [14.5,17.7] | | | 11.4 | [6.4,16.4] | 16.5 | [15.4,17.5] |
| fruit or vegetables | 42.4 | [40.8,43.9] | 35.7 | | [33.4,38.1] | | | 28.8 | [22.1,35.5] | 39.9 | [38.6,41.2] |
| bread and similar items | 7.6 | [6.9,8.4] | 8.0 | | [6.4,9.6] | | | 10.0 | [5.7,14.3] | 7.8 | [7.0,8.7] |
| sweets or chocolate | 8.2 | [7.4,9.1] | 9.2 | | [7.8,10.6] | | | 10.0 | [5.4,14.6] | 8.6 | [7.8,9.3] |
| dairy products | 9.2 | [8.3,10.0] | 11.4 | | [10.0,12.9] | | | 10.8 | [6.7,15.0] | 9.9 | [9.1,10.6] |
| does not eat between meals | 1.8 | [1.4,2.3] | 1.8 | | [1.1,2.5] | | | 3.0 | [0.6,5.4] | 1.9 | [1.5,2.2] |
| ***PHYSICAL ACTIVITY FACTORS*** | | | | | | | | | | | |
| **Number of days/week child participated in organised sports/activities (7 years)** *p<0.001* | | | | | | | | | | | |
| 3 or more days/week | 23.9 | [22.3,25.4] | 17.8 | | [16.0,19.7] | | | 10.8 | [6.1,15.5] | 21.6 | [20.3,23.0] |
| 2 days/week | 24.1 | [22.7,25.4] | 18.7 | | [16.9,20.5] | | | 11.3 | [7.4,15.2] | 22.0 | [20.9,23.2] |
| 1 day/week | 26.0 | [24.7,27.4] | 28.7 | | [26.6,30.9] | | | 23.4 | [17.3,29.4] | 26.7 | [25.6,27.9] |
| less often or not at all | 26.0 | [24.2,27.9] | 34.7 | | [32.0,37.5] | | | 54.5 | [47.6,61.5] | 29.6 | [27.8,31.4] |

|  | **Maternal psychological distress at 5 years** | | | | | | | | |
| --- | --- | --- | --- | --- | --- | --- | --- | --- | --- |
|  | **no/low distress** | | **medium distress** | | **severe distress** | | | **Total** | |
|  | **%** | **CI** | **%** | **CI** | **%** | | **CI** | **%** | **CI** |
| **Number of days/week child participated in non-organised physical activity (7 years)**  *p<0.001* | | | | | | | | | |
| 3+ days | 81.5 | [80.2,82.8] | 79.0 | [76.9,81.0] | | 77.5 | [71.4,83.6] | 80.6 | [79.3,81.9] |
| 2 days/week | 7.8 | [7.0,8.6] | 8.7 | [7.4,10.0] | | 7.2 | [3.9,10.5] | 8.0 | [7.3,8.7] |
| 1 day | 5.9 | [5.2,6.6] | 5.2 | [4.2,6.3] | | 4.8 | [1.7,8.0] | 5.7 | [5.1,6.3] |
| less often/not at all | 4.8 | [4.2,5.5] | 7.1 | [5.6,8.6] | | 10.5 | [5.6,15.4] | 5.7 | [4.9,6.4] |
| **Frequency mother participated in active play with child (7 years)**  *p<0.001* | | | | | | | | | |
| weekly | 51.1 | [49.6,52.7] | 47.9 | [45.6,50.3] | 44.7 | | [38.0,51.4] | 50.0 | [48.6,51.3] |
| Once or twice a month | 22.6 | [21.4,23.8] | 20.6 | [18.8,22.4] | 16.7 | | [12.0,21.3] | 21.8 | [20.8,22.8] |
| Less often than once a month | 14.8 | [13.8,15.8] | 16.3 | [14.6,18.0] | 16.4 | | [11.6,21.1] | 15.3 | [14.5,16.1] |
| Not at all | 11.5 | [10.4,12.5] | 15.2 | [13.6,16.9] | 22.3 | | [16.5,28.0] | 13.0 | [12.0,13.9] |
| **Transport to school (7 years)**  *p<0.001* | | | | | | | | | |
| Motorised | 51.1 | [49.5,52.7] | 46.4 | [43.8,49.1] | 37.6 | | [30.8,44.5] | 49.2 | [47.7,50.8] |
| Active (Walking/ cycling) | 48.9 | [47.3,50.5] | 53.6 | [50.9,56.2] | 62.4 | | [55.5,69.2] | 50.8 | [49.2,52.3] |
| **Regular weekday term-time bedtime (7 years)**  *p<0.001* | | | | | | | | | |
| before 19:30 | 10.1 | [9.1,11.2] | 12.4 | [10.7,14.1] | 11.3 | | [6.4,16.1] | 10.8 | [9.9,11.8] |
| 19:30-20:00 | 26.0 | [24.4,27.6] | 20.8 | [18.7,22.9] | 21.4 | | [15.9,27.0] | 24.3 | [23.0,25.6] |
| 20:00-20:30 | 36.3 | [34.8,37.7] | 33.9 | [31.7,36.1] | 33.4 | | [26.8,40.0] | 35.5 | [34.2,36.7] |
| 20:30-21:00 | 15.1 | [14.0,16.2] | 15.3 | [13.4,17.1] | 15.3 | | [10.6,20.0] | 15.2 | [14.2,16.2] |
| 21:00+ | 9.7 | [8.7,10.6] | 12.3 | [10.7,13.8] | 12.0 | | [7.9,16.1] | 10.5 | [9.6,11.4] |
| no regular bedtime | 2.9 | [2.4,3.4] | 5.3 | [4.2,6.3] | 6.6 | | [3.1,10.1] | 3.7 | [3.3,4.2] |
| **Number of hours a day child watched TV/videos (7 years)**  *p<0.001* | | | | | | | | | |
| none or up to 1 hour | 20.6 | [19.2,22.0] | 17.9 | [15.8,19.9] | 21.3 | | [15.6,27.0] | 19.8 | [18.5,21.1] |
| more than 1 hour, less than 3 | 65.5 | [64.1,67.0] | 64.7 | [62.3,67.1] | 55.5 | | [48.9,62.0] | 64.9 | [63.6,66.3] |
| 3 hours or more | 13.8 | [12.5,15.1] | 17.4 | [15.4,19.5] | 23.3 | | [17.3,29.2] | 15.2 | [14.0,16.5] |
| **Number of hours a day child used computer (7 years)**  *p<0.001* | | | | | | | | | |
| none or up to 1 hour | 66.3 | [64.6,68.0] | 60.9 | [58.5,63.4] | 57.2 | | [50.2,64.2] | 64.4 | [62.8,65.9] |
| more than 1 hour, less than 3 | 30.3 | [28.7,31.9] | 34.2 | [32.0,36.5] | 33.7 | | [26.7,40.8] | 31.6 | [30.1,33.0] |
| 3 hours or more | 3.4 | [2.9,4.0] | 4.9 | [3.6,6.1] | 9.1 | | [5.0,13.2] | 4.1 | [3.4,4.7] |
